# Mortality-Related Risk Factors in Patients with Hematologic Neoplasm Admitted to the Intensive Care Unit: A Systematic Review

**DOI:** 10.3390/curroncol32030132

**Published:** 2025-02-26

**Authors:** Jhon H. Quintana, Cesar David López-Vanegas, Giovanna Patricia Rivas-Tafurt, Leidy Tatiana Ordoñez-Mora, Heiler Lozada-Ramos, Jorge Enrique Daza-Arana

**Affiliations:** 1 Internal Medicine Specialization Program, Department of Health, Universidad Santiago de Cali, Santiago de Cali 760035, Colombia; jhon.quintana00@usc.edu.co (J.H.Q.); cesar.lopez04@usc.edu.co (C.D.L.-V.); giovanna.rivas@clinicadeoccidente.com (G.P.R.-T.); heiler.lozada00@usc.edu.co (H.L.-R.); 2 Genetics, Physiology, and Metabolism Research Group (GEFIME), Universidad Santiago de Cali, Santiago de Cali 760035, Colombia; 3 Research and Education Group GIEDCO Clínica de Occidente S.A., Santiago de Cali 760046, Colombia; 4 Health and Movement Research Group, Universidad Santiago de Cali, Santiago de Cali 760035, Colombia

**Keywords:** adult, hematologic neoplasms, intensive care units, mortality

## Abstract

Background: Hematooncology patients admitted to intensive care units (ICUs) are at high risk for mortality due to the severity of their critical illness. Such complications can develop into complex clinical management, thus signaling an urgent need to identify mortality-related factors to improve interventions and outcomes for these patients. Methods: A systematic review of studies published between 2012 and 2023 in databases such as PubMed, Scopus, and Web of Science was conducted, following the PRISMA guidelines. A meta-analysis was carried out to determine the significance of mortality-related factors. Results: In a review of twenty-four studies, it was found that invasive mechanical ventilation (IMV) was associated with an odds ratio (OR) between 2.70 and 8.26 in 75% of the studies. The use of vasopressor support had an OR of 6.28 in 50% of the studies, while pulmonary involvement by tumor had an OR of 6.73 in 30% of the studies. Sepsis showed an OR of 5.06 in 60% of the studies, and neutropenia upon admission increased mortality in 40% of the studies. Severe respiratory failure (PaO_2_/FiO_2_ < 150) had an OR of 7.69 in 55% of the studies. Additionally, ICU readmission and late admission were identified as risk factors for increased mortality. Conclusions: Mortality among hematooncology ICU patients is associated with IMV, vasopressor support, pulmonary involvement, sepsis, neutropenia, severe respiratory failure, ICU readmission, and late admission. Identifying and managing these factors in a timely manner can improve survival and the quality of care.

## 1. Introduction

Hematologic malignancies, such as leukemias, lymphomas, and myelomas, constitute a significant health problem globally, with an annual incidence ranging from 10 to 15 cases per 100,000 population/year [1]. These diseases account for 7% of all new cancer diagnoses and are responsible for approximately 10% of cancer deaths. The recent epidemiological data show that the intensive care unit (ICU) admission rate for patients with hematologic malignancies has increased by 20% in the last decade, which poses a diagnostic and therapeutic challenge due to the high rate of severe complications, including severe infections and multiorgan failure [2].

The management of patients with hematologic malignancies in the ICU also involves substantial costs. Owing to the need for intensive and prolonged therapies, these patients require 30% more resources than other critical patients. For these patients, the average length of stay in the ICU is 15 days, compared to 7 days for other critically ill patients [2].

The variability in mortality rates, ranging from 30% to >50%, reflects differences in neoplasm severity, comorbidities, and treatment strategies [3,4]. Against this background, addressing the mortality of patients with hematologic malignancies in ICUs is imperative using a comprehensive approach that combines the management of the underlying disease with the prevention and treatment of acute complications.

Recent studies, such as that of Villalobos Caballero and Espinosa Redondo et al. [5], have reported mortality rates for patients with hematologic malignancies who received chemotherapy in ICU to be between 25% and 76.5%, thus emphasizing the need for more specific interventions [5]. Pechlaner et al. [6] found that 18% of pediatric patients with hematologic oncology who were admitted to a pediatric intensive care unit as an emergency died, with invasive mechanical ventilation (IMV) and severe neutropenia being significant risk factors. Similarly, Martins and Maasdorp et al. [7] reported a mortality rate of 76.5% among adult patients with hematologic malignancies and febrile neutropenia. Septic shock and IMV were significant risk factors. Chen et al. [8] found a mortality rate of 55.9% in the ICU and identified high-grade hematologic neoplasms and organ failure as the main predictors of mortality. Aygencel Bikmaz et al. [2] highlighted that septic shock, acute kidney injury, and the need for IMV significantly increased the risk of mortality [9].

More locally, in Colombia, the associated mortality in patients treated for hematologic malignancies in the ICU reflects significant challenges and variations in outcomes. Ortiz-Martínez et al. [10] found a high prevalence of invasive fungal infections with an in-hospital mortality rate of 86%. In Medellin [11], Colombia, another study reported an ICU mortality rate of 67.52% and a total hospital mortality rate of 72.64%. The most common cause of death was septic shock (58.12%), followed by multiple organ failure (30.77%), with the main risk factors for mortality being APACHE II (odds ratio (OR) = 1.18, 95% confidence interval (CI): 1.05–1.33), use of vasopressors/nootropics (OR = 4.23, 95% CI: 1.02–17.58), and IMV (OR = 5.25, 95% CI: 1.39–19.80).

Due to the lack of consistency in the results of studies on risk factors and mortality in patients with hematologic malignancies in the ICU, it is difficult to establish standardized protocols and make clinical decisions. Therefore, a systematic review that comprehensively summarizes and analyzes the available evidence is needed. This study aims to address these research gaps through a systematic review of randomized clinical trials and cohort studies published between 2012 and 2023. The goal is to provide clarity on the current evidence and serve as a starting point to identify the most important risk factors affecting mortality in patients with hematologic malignancies in the ICU and strategies to improve their clinical outcomes.

## 2. Materials and Methods

This study followed the Cochrane Collaboration specifications for systematic reviews and criteria included in the PRISMA checklist [12]. The protocol was registered in the international prospective registry of systematic reviews, PROSPERO, with the code CRD42022350971.

Eligibility criteria were as follows:

Controlled clinical trial-type experimental studies and descriptive or analytical observational studies that respond to the established PICO (population, intervention, comparison, and outcomes) question were included.

Population: Adults aged ≥ 18 years with hematologic malignancy admitted to the intensive care unit (ICU).

Intervention: Sociodemographic, clinical, and paraclinical risk factors.

Comparation: Not applicable.

Outcomes: Mortality during ICU stay.

Articles published during the period 1 January 2012–31 December 2023 were selected. The search was performed between 7 January 2023 and 2 January 2024. The following electronic databases were searched: Elsevier, Scopus, Scielo, ScienceDirect, PubMed, LILACS, Oxford, and Springer. The search strategy included, as its main terms, “Critical Care” (MeSH) OR “Intensive Care Units” AND “Mechanical Ventilation” OR “Adults” OR “Mortality” OR “Hematologic malignancy” OR “Hematologic cancer”. Selected studies were limited to articles in English and Spanish.

### 2.1. Study Selection

A study selection process was initiated until a consensus of at least 80% was reached concerning the articles to be included. Two investigators (J.H.Q.-O. and C.D.L.-V.) performed the filtering process blindly and independently after searching the different databases. Each compiled a list of studies after analyzing the title and abstract of each article. The article was included if there was agreement between the reviewers, and if there was disagreement, a third reviewer (L.T.O.-M.), blinded to the responses of each reviewer, decided whether the article was included. The eligibility criteria were applied to analyzing the full text in the final selection. Any disagreement between authors regarding eligibility, quality, and data retrieved from the studies was resolved by consensus with another author (H.L.-R.).

### 2.2. Information Gathering Process

Data were extracted independently using a form generated in Excel Office 365 (J.H.Q.-O. and C.D.L.-V.). This form included first author and year, study design, country, sample size, age, study objective, description of risk factor-based measurement, scale used, and ICU mortality outcomes. Based on these results, the mean and standard deviations corresponding to each reported outcome (L.T.O.-M. and J.E.D.-A.) were reported, and it was confirmed that all information was consistent.

### 2.3. Assessment of the Quality of the Included Studies

The quality of the included studies was assessed using the Methodological Index for Non-Randomized Studies (MINORS) scale in an independent and blinded manner, including studies that obtained a score of ≥8 points for descriptive studies and ≥12 points for analytical studies [13].

### 2.4. Risk of Bias of Included Studies

The investigators independently assessed the risk of bias using the Risk Of Bias In Non-randomized Studies of Exposures (ROBINS-E) scale [14]. The information was compiled and analyzed using the Review Manager tool (version 5.4.1) developed by the Cochrane Collaboration. The aspects assessed included random sequence generation, allocation concealment (selection bias), blinding of participants and personnel (conduct bias), blinding of outcome assessment (detection bias), loss to follow-up (attrition bias), and selective reporting (reporting bias), all corresponding to the criteria stipulated in the ROBINS-E scale.

## 3. Results

**Figure 1:**
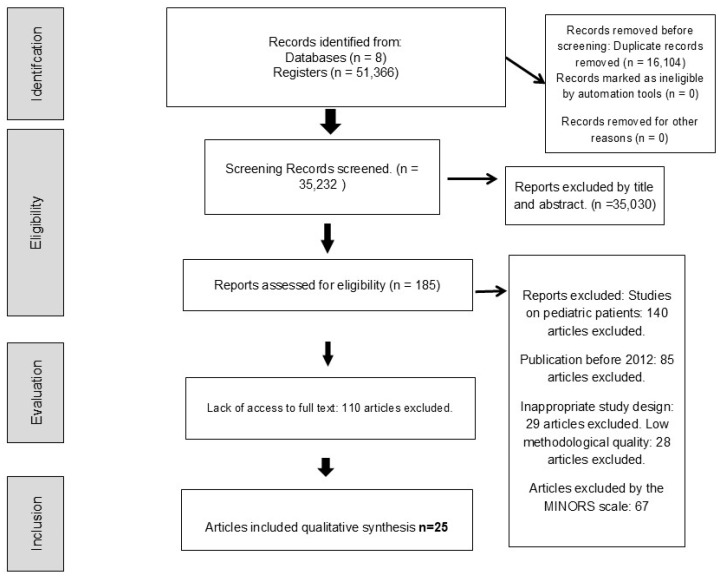
Study flow chart.

The PRISMA diagram shows the process of searching and selecting studies to include in a systematic review. A total of 51,374 studies were initially identified through databases and registries, of which 16,104 were discarded after preliminary screening for duplicates and 35,030 excluded by title and abstract, leaving 185 studies for eligibility. Finally, after a comprehensive review and the exclusion of 140 studies for various reasons—such as single-case studies, insufficient complete data, and duplicate publications—25 studies were included in the systematic review (see Figure 1).

**Summary of included studies**

The systematic review included a total of 25 studies, encompassing a wide range of publication years starting in 2012 and ending in 2023 (see Table 1). These studies vary in design, mainly prospective and retrospective observational cohorts, reflecting methodological diversity. The studies were conducted in different countries, including the USA, France, Canada, Japan, and Australia, suggesting that the findings broadly apply in different settings. Population samples from individual studies ranged from as few as 26 to >1000 patients, suggesting a meaningful analysis of clinical outcomes across settings and scales. The clinical characteristics reported included mortality and complication rates, reflecting the severity of the conditions studied and providing a compendium of evidence that could guide future clinical practice in terms of management and prevention.

### 3.1. Mortality-Related Risk Factors

It is crucial to understand the various risk factors that increase mortality in managing hematooncology patients in ICUs. IMV is one of these significant factors, as shown in multiple studies; an OR ranging from 2.70 to 8.26, reported in 75% of the studies reviewed, highlights the severity of compromised respiratory function in these patients [17,20,36].

The use of vasopressor support, which reflects severe hemodynamic instability, shows an OR of 6.280 for mortality, thus indicating a critical situation that requires intensive management, according to 50% of the studies analyzed [8,17,20]. Furthermore, pulmonary involvement by tumor also significantly increases mortality, with an OR of 6.73, present in 30% of investigations, which emphasizes the direct influence of cancer on pulmonary function and survival rates [19,36].

A particularly critical factor in this group of patients is sepsis and septic shock, both of which are common due to immunosuppression and the prevalence of neutropenia. Sepsis has an OR of 5.06 associated with mortality, reported in 60% of the studies, underscoring the severity of these infections and their impact on critically ill patients [21,23]. Neutropenia on admission to the ICU is also a significant marker of poor prognosis, increasing mortality and prolonging hospital stay, according to 40% of the sources [11,31].

Additionally, severe respiratory failure, evidenced by a PaO_2_/FiO_2_ ratio <150, has an OR of 7.69 for mortality, highlighting the importance of aggressive management of respiratory complications. This factor was reported in 55% of the studies reviewed [19,28]. Finally, late admission to the ICU was also identified as a risk factor that increased mortality, as reported in the article by Yannick Hourmant et al. [22]. Together, these factors reinforce the importance of ongoing vigilance and proactive management in the ICU to improve the chances of survival and the quality of care of hematooncology patients, whose situations are particularly complex due to the aggressive nature of their diseases and the intensive therapies required (show Table 2 and Table 3).

### 3.2. Quality Assessment

**Figure 2:**
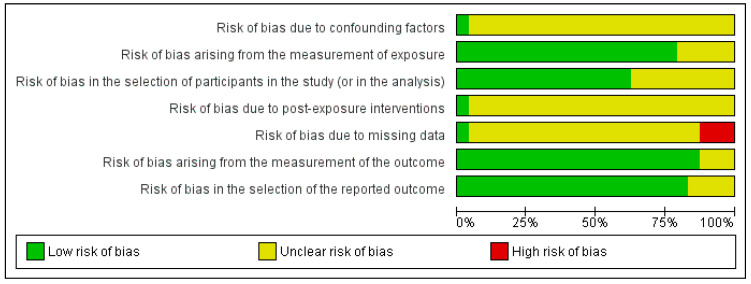
Risk of bias graph: Review authors’ judgments about each risk of bias item presented as percentages across all included studies. Own source (developed in RevMan 5.3) [38].

**Figure 3:**
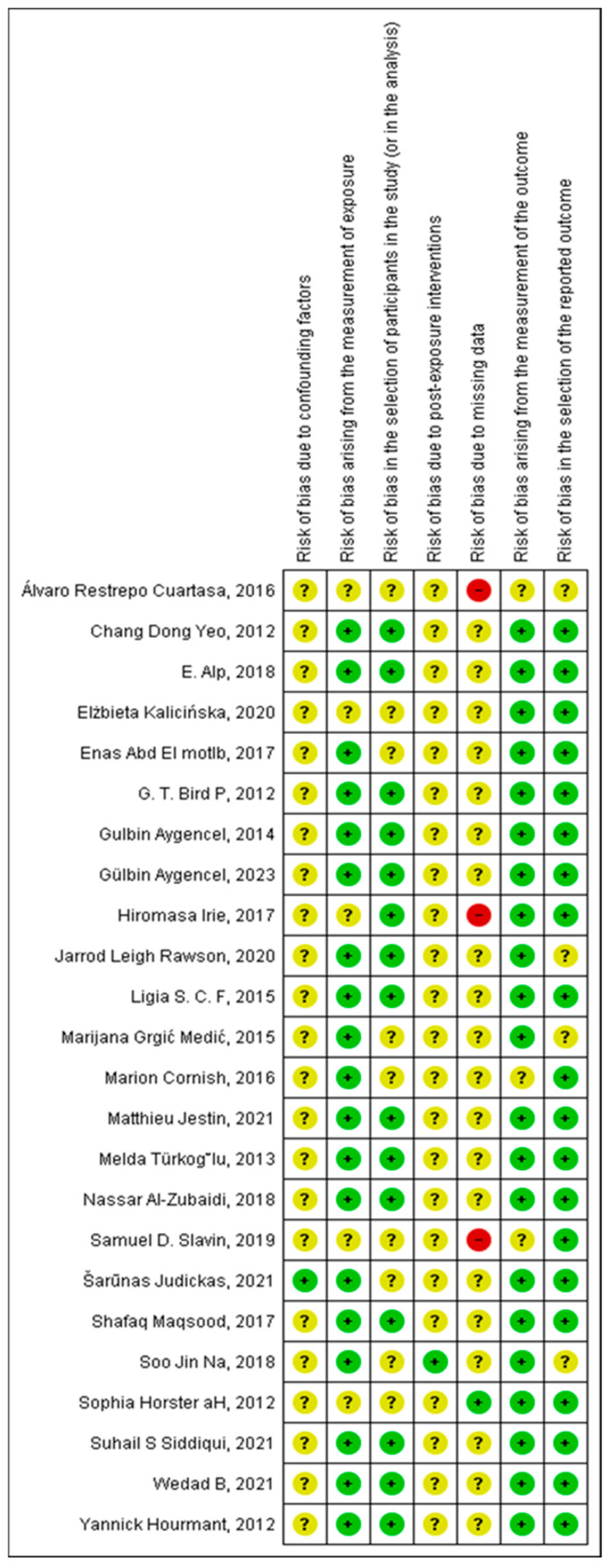
Risk of bias summary: Review authors’ judgments about each risk of bias item for each included study. Own source (developed in RevMan 5.3) [38].

A quality assessment of this study was performed using the MINORS tool, and the risk of bias was assessed using the ROBINS-E tool, which considers several risk of bias domains (see Figure 2 and Figure 3). Although most studies demonstrated a low risk of bias in key areas such as confounding (95%) and exposure measurement (85%), a significant number had an uncertain risk of bias in critical domains such as participant selection (50%), post-exposure interventions (70%), and selection of the reported outcome (15%). Such a risk of uncertain bias signals that caution is warranted when interpreting the findings. It is crucial that such uncertainties can affect the reliability of the conclusions derived from these studies. Ensuring transparency and rigor at every step of study design and reporting is pivotal to minimizing these risks, stressing the importance of improving reporting standards and methodology in future research to obtain more conclusive evidence.

## 4. Discussion

This comprehensive study identified several significant mortality-related risk factors in patients with hematologic malignancies admitted to the ICU. The findings revealed that IMV and the need for vasopressor support were the strongest predictors of poor outcomes, thus underlining their strong association with a considerable increase in mortality risk. IMV is critical because it indicates severe respiratory failure, while vasopressor support is necessary in cases of hemodynamic instability, both reflecting advanced disease states that significantly increase mortality [35]. Furthermore, severe neutropenia, which reduces the body’s ability to fight infection, and septic shock, which leads to systemic organ dysfunction, have emerged as critical conditions, significantly increasing mortality [21]. The study found that disease status at the time of ICU admission influences clinical outcomes. Specifically, the presence of neutropenia at admission and the severity of hematologic malignancy were negative prognostic factors [7]. These factors were identified through a multivariate analysis, considering clinical and demographic variables, which allowed the isolation of those elements with the greatest impact on adverse outcomes [2]. These results further emphasize how vulnerable these patients are to severe complications, thus underscoring the need for aggressive clinical and supportive management to improve survival rates and the quality of care in such a challenging setting [2,21,35]. In addition to disease status at admission, the initial symptoms leading to ICU admission were closely correlated with mortality. The most common reasons for ICU admission included severe sepsis (25.9%), pneumonia (23.7%), acute respiratory distress syndrome (ARDS) (13.3%), and multiorgan failure (11.1%) [21,22,23]. These findings highlight the critical role of early detection and management of these conditions in improving patient outcomes. Moreover, late ICU admission was identified as an independent predictor of poor prognosis. Hourmant et al. (2021) reported a mortality rate of 29.1% among patients with delayed ICU admission, which is significantly higher than those admitted earlier [22].

As mentioned above, our findings are consistent with those of previous studies that also identified IMV and vasopressor use as significant risk factors for mortality in patients with hematologic malignancies in the ICU. For example, Marion Cornish et al. [18] found that the use of IMV and the need for vasopressor therapy significantly increased 30- and 60-day mortality. Their study, based on logistic regression analysis, showed that IMV and vasopressors were critical predictors of poor outcomes, with significant ORs for short-term mortality [18]. Similarly, Grgić Medić et al. [20] reported that IMV and vasoactive treatments were mortality determinants in their 1-year analysis of patients with hematologic malignancies in the ICU, using multivariate analysis to identify these factors [20]. However, in contrast with some previous studies, such as that of Alp et al. [16], who observed that the APACHE II score and septic shock were major predictors of mortality, our analysis did not find a significant association with this score. This discrepancy could be due to differences in the populations studied, where Bird et al. [36] included a larger number of patients with bone marrow transplantation and more severe conditions, as well as variations in the methods of analysis used, which may have influenced the results [16]. Additionally, Aygencel et al. (2014) identified that patients in remission had a lower risk of mortality compared to those at initial diagnosis or relapse, with an OR of 0.113 (95% CI: 0.027–0.48; *p* = 0.003), suggesting that patients with active disease have a worse prognosis [20]. Age has also been identified as a significant prognostic factor in the mortality of patients with hematologic malignancies admitted to the ICU. Several studies have reported that the average age of ICU-admitted patients exceeded 55 years, with poorer outcomes observed in older patients. For instance, Kalicińska et al. (2015) reported an average age of 58 years among ICU patients, with a mortality rate of 70.4% [11]. Similarly, Soo Jin Na et al. (2018) found an average patient age of 63 years, with 41.7% mortality in the ICU and 53.1% hospital mortality [12]. These findings highlight the vulnerability of older patients and reinforce the need for age-specific management strategies to improve survival rates in this population. The manuscript does not mention differences in mortality based on the type of chemotherapy used (older vs. newer drugs or immunomodulators vs. immunosuppressants). However, the use of intensive chemotherapy and the need for organ support were factors associated with higher mortality, as highlighted by Villalobos Caballero and Espinosa Redondo (2022) in their analysis of patients with hematologic malignancies in the ICU [5].

The use of invasive mechanical ventilation (IMV) and vasopressors was identified as a predictor of a worse prognosis. Marion Cornish et al. (2016) found that both factors significantly increased mortality at 30 and 60 days, while Grgić Medić et al. (2015) identified IMV and vasopressor treatment as key determinants of mortality in their one-year analysis. Wedad et al. (2021) also observed a strong association between IMV and mortality in critically ill patients [3,4,6]

Additionally, pulmonary tumor infiltration and the need for urgent chemotherapy in the ICU were associated with an increased risk of mortality. Enas Abd El Motlb et al. (2017) reported that pulmonary tumor infiltration significantly increased mortality, with an OR of 6.73 (95% CI: 2.2–10.05) [5].

Furthermore, in contrast to the study by Villalobos Caballero and Espinosa Redondo [5], which reported wide variability in chemotherapy-associated mortality rates, our results suggest less variability in this specific area. This difference could be explained by the use of more standardized chemotherapy protocols and uniform clinical management in this study, thus reducing variability in outcomes [5].

One of the main strengths of this study is its rigorous methodology and use of the PRISMA guidelines for systematic reviews, which ensured that all relevant studies up to and including December 2023 were included and assessed. This provided a solid basis for reliable conclusions. This study included 25 studies with a combined sample of >10,000 patients, allowing for a more robust generalization of the results [39,40]. In addition, the geographic diversity of the studies, spanning North America, Europe, and Asia, strengthens the global applicability of the findings. The MINORS scale and the Review Manager tool developed by the Cochrane Collaboration were used to systematically assess the quality of the included studies. For example, studies with a high risk of bias were identified and excluded due to a lack of blinding and inadequate control of confounding variables. These robust methodological features minimize common limitations and yield high reliability and global applicability results in the acute care setting for hematologic malignancy patients [2,39,40].

Despite its strengths, this study faces several limitations. One significant aspect is the possible variability in defining and measuring risk factors among the studies reviewed, which could have influenced our findings. For example, differences in how IMV is measured and reported or the use of vasopressors may generate inconsistencies in the results [41]. This methodological variability may affect the comparability of studies and the accuracy of our conclusions. Furthermore, because most studies have focused on Western populations, the ability to generalize our findings to populations in other regions is limited, as seen in the study by Khwankeaw and Bhurayanontachai [42], which included a Southeast Asian population with different comorbidity profiles and clinical management. As a consequence of these limitations, the findings should be interpreted with caution.

To address these limitations, future research should aim to standardize the definitions and methods used to measure risk factors. Implementing common protocols and using validated data collection tools may improve comparison between studies. In addition, it is important to conduct prospective, multicenter studies that include greater geographic diversity. This will allow for a more global and representative view of risk factors and their effects, thereby improving the applicability of results worldwide. Ideally, these studies should also consider the inclusion of underrepresented populations and adjust analyses to control for potential confounding variables [43].

The findings of this review have important implications for clinical practice and public health policy regarding mortality in patients with hematologic malignancies in the ICU. They emphasize the need for proactive management, particularly in the early recognition and treatment of severe complications such as severe neutropenia, septic shock, and IMV. Prompt intervention can reduce mortality and improve clinical outcomes. Cornish et al. [18] demonstrated that implementing early recognition and aggressive management protocols reduced 30-day mortality by 15%. Jacobson et al. [43] observed a 20% improvement in survival with early intervention and appropriate use of vasopressors.

From a public health perspective, improving the infrastructure of ICUs and educating personnel in the specialized management of hematologic critically ill patients is crucial. The implementation of standardized protocols, such as the National Comprehensive Cancer Network) (NCCN, 2023) Protocol for the Management of Febrile Neutropenia [44], and continuing education based on international guidelines can significantly improve clinical outcomes. These findings should drive policies for continuing education of healthcare professionals and the implementation of research programs that optimize public health resources and reduce variability in clinical practice [8,20,40].

In future mortality studies in patients with hematologic malignancies in the ICU, it is crucial to include more diverse populations to improve the applicability of the findings, standardize definitions and methods for measuring risk factors, and further investigate the biological mechanisms underlying these associations. Prospective, multicenter studies are recommended to collect more robust and comparable data, focusing on assessing mortality, quality of life, and long-term outcomes. Measuring patient functionality with scales such as Eastern Cooperative Oncology Group or Karnofsky will provide a more objective assessment in this regard, while long-term outcomes could be assessed by annual follow-up of mortality and morbidity. This will facilitate the development of more accurate and effective clinical guidelines, such as the NCCN guidelines, thus optimizing the management of these highly vulnerable patients [37,43,45].

To address the identified risk factors, the implementation of specific interventions is recommended. These include the early use of broad-spectrum antibiotics in patients with severe neutropenia, optimization of IMV through pulmonary protection strategies, and aggressive management of septic shock with water resuscitation protocols and vasopressor support adapted to tissue perfusion. These interventions can reduce mortality and improve clinical outcomes, as demonstrated in previous studies [36,46].

Regarding the possibility of generating a subanalysis, no reported differences were found between the results for acute leukemia and multiple myeloma/lymphoma, nor was there any differentiation in the results due to COVID.

Study limitations may have influenced results, including heterogeneous definitions and measurements of risk factors and different analysis methods. The lack of homogeneous data may make direct comparisons between studies and extrapolating results to a larger population difficult. In future studies, it is crucial to standardize definitions and methods and use robust multivariate analyses that control for confounding variables. In addition, including more diverse populations will improve the representativeness and applicability of the results. These measures will increase the transparency and reliability of the results and thus provide a solid basis for developing more accurate and effective clinical guidelines for managing these highly vulnerable patients [47].

## 5. Conclusions

In conclusion, this systematic review study noted that mortality in patients with hematologic malignancies in the ICU is considerably high and associated with critical factors such as IMV, the use of vasopressor support, severe neutropenia, and septic shock. Such findings accentuate the complexity of managing these patients and the need for a multidisciplinary and highly specialized clinical approach. However, this study has limitations related to the heterogeneity of the data and the quality of the included studies, which could affect the generalizability of the results.

## Figures and Tables

**Table 1:** Study characteristics.

No.	Authors	Year	Country	Sample/ Population	Type of Study	Mortality (%)
1	Nassar Al-Zubaidi, et al. [15]	2018	United States	130	Observational prospective cohort	ICU: 24.8% Hospital: 45.3% 6 months: 56.7%
2	E. Alp, et al. [16]	2018	Turkey and Spain	112	Observational retrospective cohort	ICU: 73%
3	Wedad B, et al. [17]	2021	Jordan	453	Observational retrospective cohort	ICU: 48.9% Hospital:64.9%
4	Marion Cornish, et al. [18]	2016	Canada	206	Observational retrospective cohort	ICU: 45.6% 30 Days: 59.2% 60 Days: 62.6% 12 Months: 74.3%
5	Enas Abd El motlb, et al. [19]	2017	Egypt	448	Observational prospective cohort	ICU: 78%
6	Marijana Grgić Medić, et al. [20]	2015	Croatia	170	Observational prospective cohort	ICU: 53.5% ICU + IVM: 75.9%
7	Sophia Horster aH, et al. [21]	2012	Germany	90	Observational retrospective cohort	ICU: 45.6%
8	Yannick Hourmant, et al. [22]	2021	France and Belgium	1005	Observational retrospective cohort	ICU Early admission: 25.9% ICU Late admission: 29.1%
9	Hiromasa Irie, et al. [23]	2017	Japan	169	Observational retrospective cohort	ICU: 33.7% 180 days: 41.1%
10	Matthieu Jestin, et al. [24]	2021	France	26	Observational retrospective cohort	ICU: 77% Hospital: 88%
11	Šarūnas Judickas, et al. [25]	2021	Lithuania	114	Observational prospective cohort	ICU: 44.74%
12	Elżbieta Kalicińska, et al. [26]	2020	Poland	200	Observational retrospective cohort	ICU: 70.4% Hospital: 80.6%
13	Soo Jin Na, et al. [27]	2018	South Korea	175	Observational retrospective cohort	ICU: 41.7%, Hospital: 53.1%
14	Ligia S.C.F, et al. [28]	2015	Brazil	96	Observational prospective cohort	ICU: 45.8% Hospital: 64.9%
15	Jarrod Leigh Rawson, et al. [29]	2020	Australia	184	Observational retrospective cohort	ICU: 26.1%
16	Shafaq Maqsood, et al. [30]	2017	Pakistan	213	Observational retrospective cohort	ICU: 55.9% Hospital: 62.5% 30 days: 71% 1 year: 84.6%
17	Álvaro Restrepo Cuartasa, et al. [11]	2016	Colombia	117	Observational retrospective cohort	ICU: 67.52% Hospital: 72.64%
18	Suhail S Siddiqui, et al. [31]	2021	India	101	Observational prospective cohort	ICU: 48.9% Hospital: 54.3%
19	Samuel D. Slavin, et al. [32]	2019	United States	330	Observational retrospective cohort	Hospital: 53% 90 days: 65% 1 year: 70%
20	Gulbin Aygencel, et al. [33]	2014	Turkey	162	Observational retrospective cohort	ICU: 55%
21	Melda Türkoğlu, et al. [34]	2013	Turkey	68	Observational retrospective and prospective cohort	ICU: 77%
22	Chang Dong Yeo, et al. [35]	2012	South Korea	227	Observational retrospective cohort	ICU: 84.1% Hospital: 89.9%
23	G.T. Bird, P, et al. [36]	2012	United Kingdom	199	Observational retrospective cohort	ICU: 33.7% Hospital: 45.7% 6 months: 59.3%
24	Gülbin Aygencel et al. [2]	2023	Turkey	368	Observational retrospective cohort	51.4%
25	Jing Liu et al. [37]	2015	China	121	Observational retrospective cohort	ICU mortality: 60.3% 1-month mortality: 85.9% 6-month mortality: 90.9%

**Table 2:** Risk factors associated with mortality.

No.	Authors/Year	Hemato—Diagnosis Oncological	Sex—Female (%) Male (%)	Age (Years) ( $\bar{x}$)	Mortality (%)	Cause of Admission to ICU—Main Causes
1	Nassar Al-Zubaidi et al./2018 [15]	AML: 31.5% NHL: 28.5% CML: 11.5% ONH: 16.0%	46.1% 53.9%	55.5	24.8%	Severe sepsis: 25.9% Pneumonia: 23.7% SDRA: 13.3% Altered mental state: 1.6% Multi-organ failure: 11.1% Pulmonary edema: 10.4% Others: 12.6%
2	E. Alp, et al./2018 [16]	AML: 43% ALL: 21% Lymphoma 21%	38% 62%	56	73% Mortality at 30 days: 82%	Respiratory failure: 85% Need for IVM: 80%
3	Wedad B. et al./2021 [17]	Lymphoma: 13.6% Leukemia: 13.4% MM: 4.6% ONH: 0.6%	41.7% 58.3%	56.8	ICU: 48.9% Hospital:64.9%	Septic shock
4	Marion Cornish, et al./2016 [18]	Leukemia acute: 34.5% Chronic leukemia: 6.8% Lymphoma: 33.5% MM: 10.2% ONH: 15%	60.3% 39.7%	51.3	ICU: 45.6% 30 Days: 59.2% 60 Days: 62.6% 12 Months: 74.3%	Respiratory failure: 39.8% Hemodynamic instability: 37.9%
5	Enas Abd El motlb, et al./2017 [19]	CL: 6.8%	27% 73%	53.1	ICU: 78%	Pulmonary sepsis: 23% Air tract/lung invasion by tumor: 33% Coma: 16% Pulmonary embolism: 5% Cardiorespiratory arrest: 3%
6	Marijana Grgić Medić, et al./2015 [20]	AL: 41.8% CL: 9.4% Lymphoma: 31.2% Indolent lymphoma: 0.6% MM: 13.5% Others: 3.5%	48.2% 51.8%	50	UCI: 53.5%	Shock: 47.1% IRA: 37.1% FRA: 4.7% Alteration of consciousness: 5.3% Hemodynamic monitoring: 5.9%
7	Sophia Horster aH, et al./2012 [21]	Leukemia: 47.8% LAG: 50%	31.1% 68.9%	58	UCI: 45.6%	Sepsis: 28.9% IRA: 38.9% Post-interventional care: 18.9% Other reasons razones: 13.3%
8	Yannick Hourmant, et al./2021 [22]	AL: 34.3% NHL: 31.6% MM: 12.5%	39.2% 60.8%	60	ICU early admission: 25.9% ICU late admission: 29.1%	IRA: 36.9% Shock: 17.12% Sepsis: 10.4% FRA: 6.8% Coma: 22.3% Specific organ infiltration and need for chemotherapy along with organ support: 6.9%
09	Hiromasa Irie, et al./2017 [23]	Malignant lymphoma: 33.1% AML: 23.1% SMD: 16.6% ALL: 8.3% MM: 6.5% CML: 4.1% ONH:8.3%	36% 64%	63	ICU: 33.7% 180 days: 41.1%	IRA: 44.4% Sepsis: 24.8% IRA: 10.1% IC: 5.3% Neurological disorder: 5.3% Other causes: 10.1%
10	Matthieu Jestin, et al./2021 [24]	AL: 50%	50% 50%	Median: 39 years	ICU: 77% Hospital: 88%	IRA: 53% Shock: 19% Perioperative management and other less common causes
11	Šarūnas Judickas, et al./2021 [25]	AML: 40.35% NHL: 24.6% MM: 11.4% LLC: 9.65% ALL: 7% LH: 3.5% ONH: 3.5%	63% 37%	59.8	ICU: 44.74%	IRA: 42.11% Shock: 20.18% Neurological impairment: 12.28% Sepsis: 6.14% Multiorgan failure: 5.26% Post-surgical observation: 4.39% Post cardiac arrest: 1.75% Others: 7.89%
12	Elżbieta Kalicińska, et al./2015 [26]	AL: 55% AML: 46% LAG: 15% MM: 12% ONH: 18%	43.5% 56.5%	58	ICU: 70.4% Hospital: 80.6%	IRA: 85.9% Sepsis: 58.5% FRA: 51.5%
13	Soo Jin Na, et al./2018 [27]	Does not specify	33.5% 66.5%	63	ICU: 41.7%, Hospital: 53.1%	Respiratory: 35.4% Cardiovascular: 34.9% Serious infections/sepsis/septic shock: 57.1%
14	Ligia S.C.F. et al./2015 [28]	Does not specify	37% 63%	66	ICU: 45.8% Hospital: 64.9%	Septic shock: 75% IVM: 81%
15	Jarrod Leigh Rawson, et al./2020 [29]	NHL: 31.5% AML: 23.9% MM: 18.4%	31% 69%	61.9	ICU: 26.1%	Not specified
16	Shafaq Maqsood, et al./2017 [30]	NHL: 59.6% LH: 12.7% AML: 7.5% ALL: 6.6% MM: 4.2% CML: 3.3% LLC: 6.1%	29.6% 70.4%	36	ICU: 55.9% Hospital: 62.5% 30 days: 71% 1 year: 84.6%	Respiratory failure with septic shock: 29.6% Septic shock alone: 19.7% Acute respiratory failure: 13.1%
17	Álvaro Humberto Restrepo Cuartasa, et al./2016 [11]	NHL: 39.32% AML: 18.80% ALL: 13.68% MM: 10.26% LH: 8.55% CML: 5.98% LLC: 2.56%	47.86% 52.14%	49.78	ICU: 67.52% Hospital: 72.64%	Respiratory failure: 42.74% Sepsis/septic shock: 36.75%
18	Suhail S Siddiqui, et al./2021 [31]	ALL: 18.8% AML: 28.7% NHL: 37.6% LH: 5.9% CML: 1% NCP: 7.9%	47.5% 52.5%	41.44	ICU: 48.9% Hospital: 54.3%	Acute respiratory failure: 36.6% Septic shock: 30%
19	Samuel D. Slavin, et al./2019 [32]	100% AML	59.1% 40.9%	69 (60–90)	Hospital: 53% 90 days: 65% 1 year: 70%	Respiratory Failure: 40% Septic Shock: 29% Neurological Compromise: 9%
20	Gulbin Aygencel, et al./2014 [33]	AML	41.4% 58.6%	61	ICU: 55%	Sepsis or septic shock: 66.7% IRA: 63.6%
21	Melda Türkoğlu, et al./2013 [34]	Leukemia: 60%	31% 69%	45	ICU: 77%	Pneumonia: 80% Sepsis of non-pulmonary origin: 10% Unknown cause: 10%
22	Chang Dong Yeo, et al./2012 [35]	AML: 51.5% ALL: 15% CML: 5.3% MM: 13.7% SMD: 6.6% Malignant lymphoma: 7.9%	38.3% 61.7%	51.2	ICU: 84.1% Hospital: 89.9%	IRA: 36.6% Septic Shock: 30% Gastrointestinal: 12% Neurological: 6% Renal/Metabolic: 6% Cardiovascular Complications: 5% Subsequent Cardiac Arrest: 4% Other Causes: 1%
23	G.T. Bird, P, et al./2012 [36]	Does not specify	43.7% 56.3%	58	ICU: 33.7% Hospital: 45.7% 6 months: 59.3%	Respiratory failure: 33.7% Sepsis: 21.1% Postoperative: 20.1% Kidney failure: 8.0% Heart problems: 7.0% Gastrointestinal problems: 3.0% Neurological problems: 2.5% Others: 1.5% Unknown: 3.0%
24	Gülbin AYGENCEL et al./2023 [2]	AML:23.3%	36.7% 63.3%	Median: 58 years	ICU: 51.4%	Sepsis and septic shock (75.3%) Respiratory failure (68.2%)
25	Jing Liu et al./2015 [37]	AML: 52.0% ALL: 13.2% Malignant lymphoma: 19.8%	28.1% 71.9%	48 years (range 16–82 years)	Mortality in ICU: 60.3% Mortality at 1 month: 85.9% Mortality at 6 months: 90.9%	Acute Respiratory Failure: 57.9% Shock: 11.6% Coma: 17.3%

Abbreviations: AML: Acute Myeloid Leukemia, NHL: Non-Hodgkin Lymphoma, CML: Chronic Myeloid Leukemia, ONH: Other Hematological Neoplasms, ALL: Acute Lymphoid Leukemia, CLL: Chronic Lymphocytic Leukemia, MM: Multiple Myeloma, AL: Acute Leukemia, CL: Chronic Leukemia, LAG: High Grade Lymphoma, MDS: Myelodysplastic Syndrome, PCN: Plasma Cell Neoplasia, ICU: Intensive Care Unit, ARDS: Acute Respiratory Distress Syndrome, IMV: Invasive Mechanical Ventilation, ARF: Acute Respiratory Failure, AKI: Failure Acute Renal.

**Table 3:** Characteristics of the studies included in the systematic review with measurements of association with mortality-related risk factors.

Author/Year	Mortality-Related Risk Factors	Measurements of Association:
1. Nassar Al-Zubaidi et al./2018 [15]	IVM 2. Allogeneic bone marrow transplantation	OR = 19.0; 95% CI: 3.1–117.4; *p* = 0.001 2. OR = 10.9; 95% CI: 1.8–66.9; *p* = 0.01
2. Alp et al./2018 [16]	APACHE II score ≥25 Septic shock 3. Respiratory failure	OR = 35.20; 95% CI: 4.71–263.25; *p* = 0.001 2. OR = 8.71; 95%CI: 1.34–56.38; *p* = 0.023 3. OR = 10.55; 95% CI: 1.05–105.73; *p* = 0.045
3. Wedad et al./2021 [17]	Mechanical Ventilation 2. APACHE II 3. Thrombocytopenia 4. Positive cultures 5. Elevated bilirubin level 6. Elevated lactic acid level	OR = 2.109; 95% CI: 1.682–2.643; *p* = 0.0001 2. OR = 1.054; 95% CI: 1.039–1.069; *p* = 0.0001 3. OR = 1.611; 95% CI: 1.285–2.020; *p* = 0.0001 4. OR = 1.632; 95% CI: 1.301–2.048; *p* = 0.0001 5. OR = 1.940; 95% CI: 1.366–2.757; *p* = 0.0002 6. OR = 1.355; 95% CI: 1.009–1.820; *p* = 0.0436
4. Marion Cornish et al./2016 [18]	1. Use of IMV Need for vasopressor therapy 2	OR = 2.21; 95% CI: 1.05–4.63; *p* = 0.036 for 30 days OR = 2.14; 95% CI: 1.01–4.55; *p* = 0.047 for 60 days
5. Enas Abd El motlb, et al., 2017 [19]	Functional Status (ECOG 3–4) Recurrence/Progression of Cancer PaO_2_/FiO_2_ < 150 Pulmonary Tumor Involvement Use of Vasopressors	OR = 2.49; 95% CI: 1.35–4.60 OR = 9.31; 95% CI: 4.18–21.24 OR = 2.47; 95% CI: 1.344–4.68 OR = 6.73; 95% CI: 2.2–10.05 OR = 3.39; 95% CI: 1.73–6.44
6. Marijana Grgić Medić et al., 2015 [20]	Use of IVM Use of vasoactive treatment	OR = 2.710; 95% CI: 1.433–5.128; *p* = 0.002 OR = 6.280; 95% CI: 2.689–14.705; *p* = <0.001
7. Sophia Horster et al./2012 [21]	Respiratory failure Norepinephrine ≥ 3 mg/h Renal support therapy SAPS II ≥ 54	HR: 2.50; 95% CI: 1.01–6.23; *p* = 0.048 HR = 2.96; 95%CI: 1.61–5.42; *p* = <0.0001 HR = 1.93; 95% CI: 1.08–3.46; *p* = <0.026 HR = 1.02; 95% CI: 1.00–1.04; *p* = 0.027
8. Yannick Hourmant, et al./2021 [22]	1. Age 2. Allogeneic stem cell transplantation 3. Hepatic comorbidity 4. Poor Performance Status (PS) 5. SOFA Scoring	1. HR: 1.01 per year; 95% CI: 1.01–1.02 2. HR: 1.54; 95% CI: 1.20–1.98 3. HR: 1.42; 95% CI: 1.08–1.87 4. HR: 1.38; 95% CI: 1.12–1.72 5. HR: 1.14 per point; 95% CI: 1.12–1.17
9. Hiromasa Irie et al./2017 [23]	1.IVM 2. A high SOFA score in the first 24 h	OR = 8.96; 95% CI: 3.67–21.9; *p* = 0.00 2. OR = 1.25; 95% CI: 1.11–1.40; *p* = 0.001
10. Šarūnas Judickas et al./2015 [25]	qSOFA ≥ 2 Increase in SOFA score in the first 48 h in the ICU IVM on the first day in the ICU Need for colistin therapy in the ICU Arterial pH upon admission to the ICU	OR = 4.403; 95% CI: 1.376–14.081; *p* = 0.0125 OR = 11.171; 95% CI: 2.072–60.226; *p* = 0.0156 OR = 6.157; 95% CI: 1.867–20.308; *p* = 0.0028 OR = 11.037; 95% CI: 2.673–45.572; *p* = 0.0009 OR: 0.392; 95% CI: 0.201–0.7620; *p* = 0.0058
11. Elżbieta Kalicińska et al./2015 [26]	Prolonged stay in ICU Acute respiratory failure Need for renal replacement therapy	OR = 6.98; 95% CI: 1.38–35.33; *p* = 0.02 OR = 5.35; 95% CI: 1.01–28.46; *p* = 0.04 OR = 8.75; 95% CI: 1.23–62.11; *p* = 0.03
12. Soo Jin Na, et al./2018 [27]	SAPS3 (Simplified Acute Physiology Score 3) Use of IVM	OR = 1.05; 95% CI: 1.01–1.08; *p* = 0.006 OR = 2.41; 95% CI: 1.05–5.55; *p* = 0.039
13. Ligia S.C.F. Rabello1 et al./2015 [28]	IVM in the ICU Septic Shock in ICU Admission Performance Status	OR = 12.74; 95% CI: 3.60–45.07; *p* = 0.001 2. OR = 5.52; 95% CI: 1.92–15.85; *p* = 0.002 3. OR = 3.00; 95% CI: 1.07–8.42; *p* = 0.037
14. Jarrod Leigh/2020 [29]	Treatment induction Post-transplant treatment APACHE II scores APACHE III scores	Induction treatments (*p* = 0.0001) worse Prognosis Post-transplantation treatment (*p* = 0.041) worse prognosis APACHE II prediction (*p* = 0.002) was used as a predictor of mortality. 4. APACHE III prediction (*p* = 0.0001) was used as a predictor of mortality
15. Shafaq Maqsood et al./2017 [30]	Need for IVM Multiorgan failure	Association between IMV and mortality (*p* = 0.001) 2. Association between multiorgan failure and mortality (*p* = 0.001)
16. Álvaro Humberto Restrepo Cuartasa, et al./2016 [11]	APACHE II 2. Use of vasopressors/inothropics 3. IMV	OR = 1.18; 95% CI: 1.05–1.33; *p* = 0.005 OR = 4.23; 95% CI: 1.02–17.58; *p* = 0.047 OR = 5.25; 95% CI: 1.39–19.80; *p* = 0.014
17. Suhail S Siddiqui et al./2021 [31]	Presence of neutropenia upon admission to ICU Need for MV within 24 h of ICU admission	OR = 2.728; 95%CI: 1.077–6.912, *p* = 0.034 OR = 4.621; 95%CI: 1.230–17.357, *p* = 0.023
18. Samuel D. Slavin, et al./2019 [32]	Number of life support therapies A therapy Two or more therapies 2. Baseline performance status	1. OR 2.57; 95%CI: 0.66–10.06, *p* = 0.174 OR 12.39; 95% CI: 3.10–49.48, *p* = 0.001 2. OR 2.76; 95%CI: 1.24–6.12, *p* = 0.013
19. Gülbin Aygencel, et al./2014 [33]	Cancer remission status on admission to ICU APACHE II score Sepsis/septic shock during ICU stay Vasopressor requirement	OR 0.113; 95% CI: 0.027–0.48; *p* = 0.003 OR 1.12; 95%CI: 1.032–1.215; *p* = 0.007 OR 8.94; IC95%28–35; *p* = 0.002 OR 16.84; 95%CI: 3.98–71.24; *p* = 0.0001
20. Melda Türkoğlu, et al./2013 [34]	Presence of multiorgan failure Use of NIV	OR = 6.61; 95%CI: 1.04–41.93, *p* = 0.045 OR = 0.04; 95%CI: 0.045, *p* = 0.001
21. Chang Dong Yeo, et al./2012 [35]	1. Acute leukemia 2. Need for invasive mechanical ventilator 3. Use of inotropic agents 4. APACHE II score	1. OR: 3.40, 95%CI: 1.25–9.26, *p* = 0.017 2. OR: 4.75, 95%CI: 1.40–16.12, *p* = 0.012 3. OR: 3.83, 95% CI: 1.40–10.50, *p* = 0.009 4. OR: 1.12 (per unit increase in APACHE II score), 95% CI: 0.83–0.95, *p* = 0.001
22. G.T. Bird, P, et al./2012 [36]	IVM Failure of ≥2 organ systems. organ systems	OR = 3.03; 95% CI: 33–6.90 2. OR = 5.62; 95% CI: 2.30–13.70
23. Gülbin Aygencel et al./2023 [2]	SOFA score upon admission to ICU IVM support during ICU stay Development of septic shock during ICU stay Development of acute kidney injury during ICU stay	OR: 1.281; 95% CI: 1.082–1.517; *p* = 0.004 OR: 23.118; 95%CI: 6.577–81.263; *p* = 0.0001 OR: 17.123; 95%CI: 4.954–59.18; *p* = 0.00013 OR: 48.284; 95%CI: 12.232–190.594; *p* = 0.0001
24. Jing Liu et al./2015 [37]	IMV 2. APACHE II score at admission 3. SOFA score unchanged/increased	1. OR: 14.167; 95% CI: 3.007–66.748; *p* = 0.001 2. OR: 1.241; 95% CI: 1.093–1.408; *p* = 0.001 3. OR: 9.031; 95% CI: 1.061–76.855; *p* = 0.044

Abbreviations: OR: Odds Ratio, CI: Confidence Interval, *p*: *p*-Value, HR: Hazard Ratio, APACHE: Acute Physiology and Chronic Health Evaluation, ECOG: Eastern Cooperative Oncology Group, PaO_2_/FiO_2_: Ratio of arterial blood oxygen partial pressure to inspired oxygen fraction, SAPS: Simplified Acute Physiology Score, SOFA: Sequential Organ Failure Assessment, qSOFA: Quick Sequential Organ Failure Assessment, NIV: Noninvasive Ventilation, IMV: Invasive Mechanical Ventilation.
